# Magnetic structure and Magnetic transport Properties of Graphene Nanoribbons With Sawtooth Zigzag Edges

**DOI:** 10.1038/srep07587

**Published:** 2014-12-23

**Authors:** D. Wang, Z. Zhang, Z. Zhu, B. Liang

**Affiliations:** 1 Institute of Nanomaterial & Nanostructure, Changsha University of Science and Technology, Changsha 410114, China; 2 School of Automotive & Mechanical Engineering, Changsha University of Science and Technology, Changsha 410114, China

## Abstract

The magnetic structure and magnetic transport properties of hydrogen-passivated sawtooth zigzag-edge graphene nanoribbons (STGNRs) are investigated theoretically. It is found that all-sized ground-state STGNRs are ferromagnetic and always feature magnetic semiconductor properties, whose spin splitting energy gap *E_g_* changes periodically with the width of STGNRs. More importantly, for the STGNR based device, the dual spin-filtering effect with the perfect (100%) spin polarization and high-performance dual spin diode effect with a rectification ratio about 10^10^ can be predicted. Particularly, a highly effective spin-valve device is likely to be realized, which displays a giant magnetoresistace (MR) approaching 10^10^%, which is three orders magnitude higher than the value predicted based on the zigzag graphene nanoribbons and six orders magnitude higher than previously reported experimental values for the MgO tunnel junction. Our findings suggest that STGNRs might hold a significant promise for developing spintronic devices.

Spintronic devices (STDs), using spin instead of charge as an information carrier, have recently attracted tremendous attention within the scientific community. Due to unique electronics as well as magneto-electronics properties, such as a weak spin-orbital coupling and long spin correlation length for electrons, which are some key features for developing STDs^1^,^2^,^3^, the two-dimensional planar graphene and the corresponding quasi-unidimensional graphene nanoribbons (GNRs) have been extensively studied^1^,^2^,^3^,^4^,^5^,^6^,^7^. Especially for zigzag graphene nanoribbons (ZGNRs), one predicts that they will play an important role in spintronic applications. Ground-state ZGNRs exhibit ferromagnetically (FM) ordered states at each edge individually due to the unsaturated π electron existing for each edge carbon atom but antiferromagnetic (AFM) coupling between opposite edges, leading to a spin-polarized semiconducting behavior with zero net spin^4^, thus their applications in STDs are severely limited. Heretofore, many effective approaches, such as edge modifications^8^, doping^9^, introducing topologic defects^10^, and applying an external electrical field^4^ or magnetic field^5^, have been proposed to break the spin degeneracy and stabilize ferromagnetic (FM) state in ZGNRs, achieving metallic or half- metallic features accordingly. Based on these ways, some important phenomena are found and promising STDs, such as giant magnetoresistance devices and conductance switchers^5^, spin-filtering devices^6^, bipolar spin diodes^6^,^7^, spin-polarized current amplifiers^7^, and bipolar field-effect spin-filtering devices^11^, are designed theoretically.

However, in fact, the realistic applications of the ZGNR magnetism and the above methods to obtain a favorable magnetic ordering might not be feasible. Generally, a very large electrical field is required to split the spin-degenerated band structures and achieve a half-metallicity experimentally. The theoretical predicted magnetic moment for edge carbon atoms is not very strong^12^, so that the spin-polarized states of the ZGNRs become unstable to be transformed to the spin-unpolarized state in the presence of ballistic current through the ZGNRs^13^,^14^ or at finite temperatures^15^. It was estimated that the magnetic states can only be stabilized at temperatures *T* < 10*K*^16^,^17^, and become paramagnetic(PM) behaviors as *T* increases, which has been confirmed experimentally^18^. And also, as reported in previous works^5^,^6^,^7^, STD effects based on ZGNRs essentially occur for geometrically symmetrical ZGNRs with respect to the axis due to the intrinsic transmission selection rule of the wave function of spin subbands near the Fermi level, but not for geometrically asymmetrical ZGNRs. Particularly, realizing the specific magnetic device features needs the externally magnetic field simultaneously applied on left and right ZGNR electrodes to create the [1,-1] magnetic configurations^5^,^6^,^7^, however, it is difficult to limit two magnetic fields with the opposite direction in two local nano-scale regions, which might exceed those available experimentally^19^. Additionally, heteroatom doping would influence the mobility and spin correlation length of carriers, even resulting in a spin flip, and edge modification might weaken the geometrical structure stability. Therefore, designing graphene-based systems to have a large ground-state magnetic moment and become experimentally feasible STDs remains a challenge.

In this paper, we present investigations on magnetic structure and magnetic transport properties for the graphene nanoribbons (GNRs) with sawtooth (ST) zigzag edges (STGNRs)^20^ and passivated by mono- hydrogen atom^21^, which is related to modified-edge GNRs^22^,^23^. It is found that at a FM ground state they are all a magnetic semiconductor. In particular, unique band overlap pattern for two electrodes and its particular sensitivity to a switching magnetic field lead to the dual spin-filtering effect with the perfect (100%) spin polarization and high-performance dual spin diode effect with a rectification ratio about 10^10^, as well as a highly effective spin-valve device feature with a giant magnetoresistace (GMR) value approaching 10^10^% can be observed.

## Results

**Figure 1:**
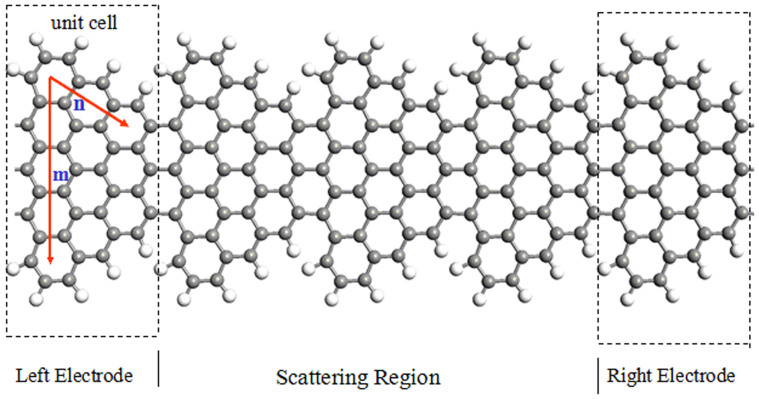
The schematic diagram of geometrical structures and the constructed device model for STGNRs(m, n), the dashed box indicates the unit cell, and (m, n) denotes the size of a unit cell.

Fig. 1 illustrates the schematic structure of STGNRs (m, n), and the rectangle box drawn with a dotted line denotes a unit cell. STGNRs can be view as the armchair graphene nanoribbons (AGNRs) being tailored or introducing defects at their edge to form zigzag edges with a large sawtooth periodically. Current nano-size lithographic techniques have provided the possibility for cutting or patterning graphene into well-defined geometric structures with atom precision, for example, the controlled formation of sharp zigzag edges in GNRs has been recently fabricated experimentally^24^. The dangling bond on each edge carbon atom for STGNRs (m, n) is all saturated by single hydrogen (H) atom in order to retain the sp^2^ hybridization of carbon atoms. Here, two integers, m and n, are used to represent the unit-cell size of STGNRs, which are the number of hexagonal rings along the *m* and *n* directions, respectively, as shown in Fig. 1, corresponding to the width of nanoribbon and the length of a large sawtooth at edge. It is important to note that to form a periodic nanoribbon structure, *n* must be an odd number and equal to or bigger than 3, i.e., *n* ≥ 3, and both *m* and *n* satisfy *m* − *n* ≥ 1 to assure STGNRs being the same width for the whole nanoribbon. Further, we construct device models by the STGNRs to investigate magnetic transport behaviors, where a device is divided into three regions: left electrode, right electrode, and the scattering region (the device region). Each electrode is represented by a unit cell of STGNR along the axis and descried by self-energies, and the scattering region is involved in the self-consistent cycle for calculations and takes into account the interface coupling and screening layer effects.

**Figure 2:**
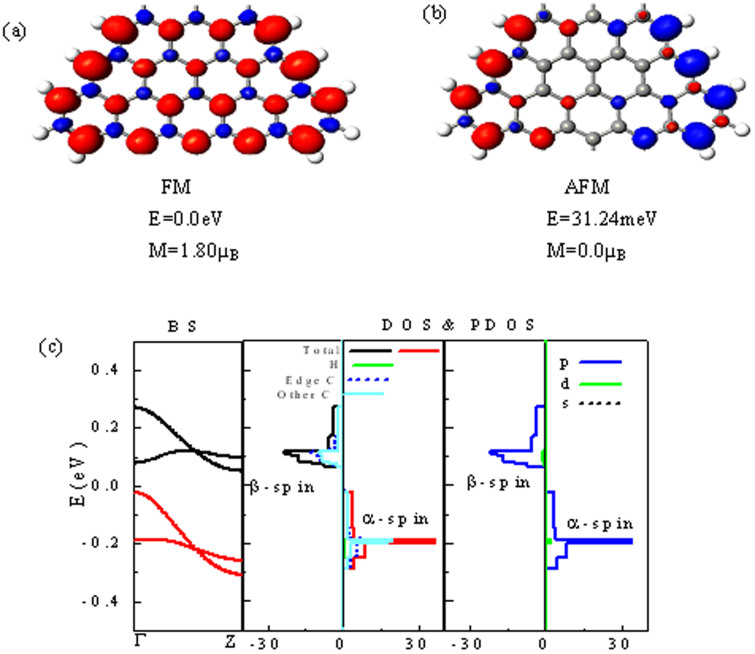
Electronic and magnetic structures of STGNR(5,3). Isosurface plots of the spin density (∇ρ = ρ_α_ − ρ_β_) of the FM ground state (a) and AFM state (b) for an optimized H-STGNR(5,3). Values for red (α-spin) and blue (β-spin) isosurfaces are ±0.01|e|/Å^3^, respectively. (c) The band structure (BS), density of states (DOS), and projected density of states (PDOS) for STGNR(5,3). PDOS includes the atom-PDOS and orbital- PDOS.

We firstly investigate electronic and magnetic structures of STGNRs with STGNR(5,3) as example. The isosurface plots for the spin polarized density (∇ρ = ρ_α_ − ρ_β_) of the FM and AFM states are shown in Fig. 2(a) and (b), respectively, where ρ_α_ and ρ_β_ denote the electron density of majority spin (α) (red) and minority spin (β) (blue), respectively. For the FM state as shown in Fig. 2(a), all the carbon atoms display spin-polarized states, specially, there exists a spin parallel coupling between both edges. While for the AFM state, as shown in Fig. 2(b), STGNR displays FM ordering at each edge carbon atoms individually, but AFM coupling between both edges, similarly to ground-state ZGNRs. Our calculations show that the FM state is a ground state, its total energy is found to be 31 meV per unit cell lower than the AFM state. These results are easy to be understood. For graphene, the spin of the A and B sublattices is antiparallel. In a STGNR, the edge carbon atoms always belong to the same sublattice, A sublattice, except for carbon atoms with the armchair bond at corners. The polarization of carbon atoms in the A sublattice, especially at the edges, is much stronger than that in the B sublattice, which is actually due to the unsaturated bond of those atoms, even after they are hydrogenated. Upon the strong interactions between the carbon atoms, the inside atoms are also spin-polarized. For a STGNR (m, n), the number of A-type carbon atoms and B-type carbon atoms is of unbalanced, they are 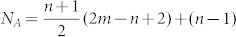 and 
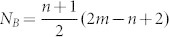
 per unit cell, respectively. Thus the difference of the number for two types of carbon atoms is *N_A_-N_B_* = *n*-1, independent of *m*. According to Lieb's theorem, total net spin magnetization of a graphene structure is 
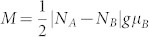
, where *g* ≈ 2 for the electron, *μ_B_* is the Bohr magneton. Applying this rule, we can obtain that the magnetic moment is 2µ_B_ for STGNR(5, 3). Our calculations on Mulliken Population involving the spin polarization show that the magnetic moment for STGNR(5, 3) is about 1.85µ_B_ per unit cell, which basically agrees with Lieb's theorem. This slight smaller value is because the interactions between edge carbon atoms and H atoms induce a rearrangement of electron spins of carbon atoms^25^.

The spin-resolved band structure (BS), the density of states (DOS), and the projected density of states (PDOS) for STGNR(5, 3) at the FM ground state are exhibited in Fig. 2 (c). The middle and right panels display the atom-PDOS and orbital-PDOS, respectively. As can be seen, α- and β-spin subbands are prominently split, but no subband (or DOS) crosses (or touches) the Fermi level, and the conduction and valance bands nearest to the Fermi level belong to β- and α-spin states, respectively. This indicates that STGNR(5, 3) features the properties of bipolar magnetic semiconductors (BMS)^11^ with a well-defined spin splitting energy gap *E_g_*, ~75 meV. The BMS is an important material for realizing bipolar spin-filtering (BSF) devices^11^. The middle and right panels for the PDOS suggest that the DOS around the Fermi level is mainly derived from *p* (π) orbital (the *s*- and *d*-orbital PDOS is negligible small) of C atoms(at both edges and others).

**Figure 3:**
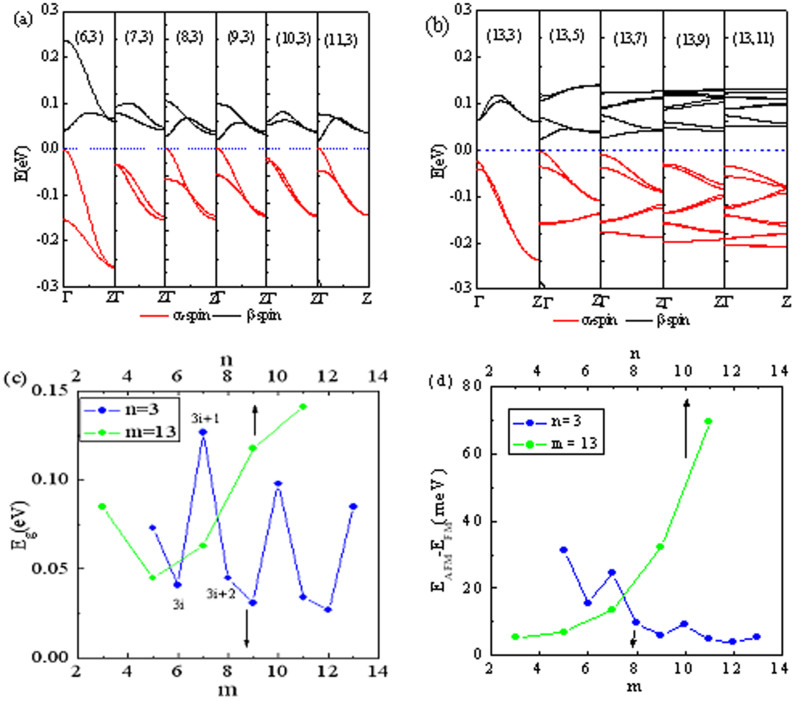
Size effects for STGNRs(m,n). (a), (b), (c), and (d) show the change of the band structures, spin splitting energy gap, and energy difference *ΔE_mag_* between the AFM state and the FM state with geometrical parameters *m* and *n*, respectively

To find the size effects, the band structure and spin splitting energy gap as a function of geometrical parameters, *m* and *n*, corresponding to the width of nanoribbon and the length of a large sawtooth at edge for STGNRs, are shown in Fig. 3(a–c). It is clear that the magnetic semiconducting behaviors for all STGNRs are always preserved regardless of the value of *m* and *n.* Interestingly, the spin splitting energy gap *E_g_* changes periodically with the value of *m* by 3 as a period, and satisfies 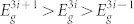 (*i* is a positive integer), similarly to the case of AGNRs for the gap alteration with width. With increasing the value of *n*, the spin splitting energy gap *E_g_* drops at n = 5 then rises sharply.

For realistic applications of magnetic materials, the magnetic stability is an important aspect that needs to be considered. As we know, the paramagnetic response of a system is described by the Pauli susceptibility which depends only on the electronic density of states at the Fermi level (Stoner instability)^26^. STGNRs have almost zero DOS at the Fermi level at the FM ground-state as shown in Figs. 2 (c), thus giving rise to a higher ground-state magnetic stability for STGNRs than that for the FM state of ZGNRs with finite DOS at the Fermi level^5^. To further investigate a thermal stability, the energy difference *ΔE_mag_* between the AFM and FM states as a function of *m* and *n* is drawn in Fig. 3(d). As mentioned above, the FM is the ground state (GS) with a lowest energy, while the AFM is the second-lowest energy state (GS+1), and the nonmagnetic state possesses a highest energy (GS+2)(not exhibited here). Generally, the thermal stability of materials is regarded to be related to *ΔE_mag_*. As can be seen, *ΔE_mag_* decreases with increasing *m*, but arises monotonically with increasing *n,* this is easy to be understood. When the value of *m* increases to form a wider ribbon, the edge magnetic coupling (exchange interaction) is weakened accordingly to display pristine AGNR properties partially. Conversely, increasing *n* means increasing the length of zigzag edges and in turn decreasing their spacing, so that the two-edge magnetic coupling is strengthen unambiguously. The thermal stability is usually quantified by the Curie temperature *T_c_* based on the mean-field theory and Heisenberg model^27^, *T_c_* = 2Δ/3k_B_, whereΔ is the energy required to flip one spin to the GS+1 state. We can obtain Δ = 12.24 meV for STGNR (4, 3), leading to *T_c_ ~* 95 *K*. It is almost 10 times as large as ZGNRs by the experiment value *T_c_* < 10*K*^16^,^17^, but still much below room temperature.

**Figure 4:**
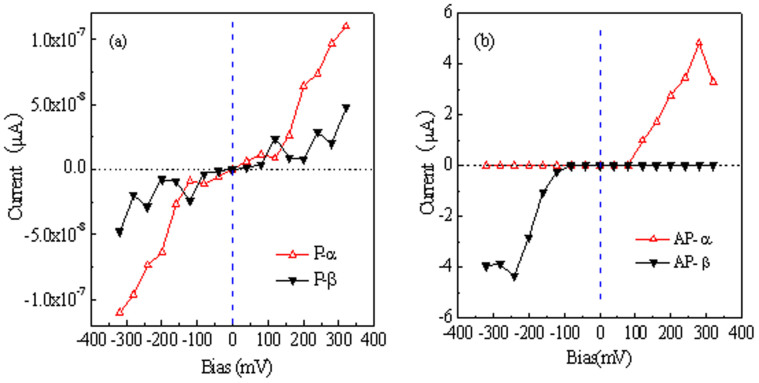
The spin-resolved I-V characteristics in P (a) and AP (b) configurations for STGNR(5,3). In P configuration, the electron tunneling channels for both α- and β-spin states are almost blocked off completely, but in AP configuration, the bias-polarity-dependent spin-polarized current shows up.

To develop future STDs based on STGNRs, it is highly desirable to fully understand their intrinsic magnetic transport properties under bias. The constructed device model is shown in Fig. 1, as stated above. Here, two types of magnetic configurations are considered, respectively : (1) P configuration, namely, whole device is taken as a FM ground state, and the spin ordering for two electrodes in parallel points to the same direction. α- and β-spin states are major and minor spin components, respectively. (2) AP configuration, i.e., an externally switching magnetic field is applied perpendicular to the plane of the right electrode to switch its spin ordering antiparallel to the left electrode, namely, the left electrode is still α-spin polarized but the right one is turned to be β-spin polarized, in order to demonstrate the response of STGNRs to an application of the magnetic field. Furthermore, the spins across the magnetic domain wall in the scattering region is set in a collinear case for calculations. We still take STGNR(5,3) as example, and for the corresponding device, the spin-resolved I-V characteristics in P and AP configurations are manifested in Figs. 4 (a) and (b), respectively. Obviously, several important features can be visible: (1) In P configuration, the electron tunneling channels for both α- and β-spin states are almost blocked off completely, the negligible small currents (< 5 × 10^−7^ µA)in a region of interest can be observed. (2) In AP configuration, the unidirectional nature of the spin-polarized current shows up, namely, the α-spin channel is only opened under positive bias but suppressed fully under negative bias, while for the β-spin channel, it is just opposite. These mean that the STGNRs can act as a dual spin filter and a dual spin diode in AP configuration. (3) Making a comparison on currents between P and AP configurations, one can find that there is a much larger current (several µA) in AP configuration than that (< 5 × 10^−7^ µA)in P configuration, which implies that a switching magnetic field features a tremendous tuning effects on the spin transport and thus the giant magnetoresistance (MR) effect can be expected reasonably.

**Figure 5:**
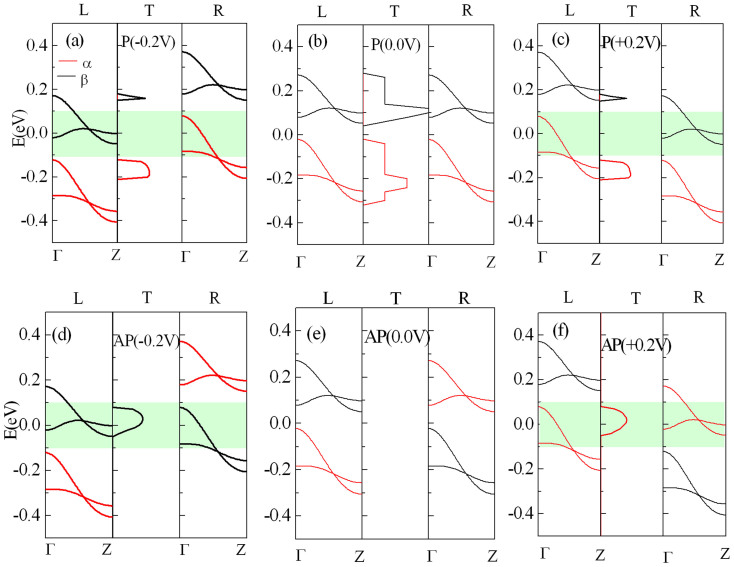
The relation of the transmission spectrum and electrode band structures for STGNR(5,3) under several typical biases, 0.0 and ±0.2 V. (a) – (c) For P configuration.(d) – (f) For AP configuration. The region highlighted with green represents the bias window.

To understand the origin of distinctive transport behaviors, the relation of the transmission spectrum and electrode band structures under several typical biases, 0.0 and ±0.2 V, are displayed in Fig. 5(a) – (f), in which figures (a) – (c) and (d) – (f) correspond the P configuration and AP configuration, respectively. In each figure, the left, middle, and right panels show the band structure of the left electrode, the transmission spectrum of the device, and the band structure of the right electrode, respectively. To obtain a spin-dependent current, the band overlap is necessary, namely, the bands for the same spin state in two electrodes form an overlapping region in the bias window (BW) and make the electronic band-to-band tunneling available for carrying current. In P configuration, at zero bias, as shown in Fig. 5(b), the overlap of β- and α- spin bands in both electrodes above and below the Fermi level leads to two transmission peaks, respectively, but they do not contribute to the realistic electron transmission due to this transmission gap occurring around the Fermi level. When this device is negatively (positively) biased, the bands of the left and right electrodes are driven to move downward (upward) and upward (downward), respectively. Until - 0.2 (+0.2) V, as shown in Fig. 5(a) ((c)), no spin band overlap appears for the same spin state of two electrodes within the BW, resulting in the BW always lying in an transmission gap. And from then onwards, the transmission gap will increases monotonically with bias and the BW remains in between. Therefore, almost zero current arises in a device for P configuration regardless of the bias polarity and spin components. But for AP configurations, the situation is greatly changed. The applied switching magnetic field turns the role of α- and β-spin states in the right electrode to be exchanged each other, namely, minor and major spin components for α- and β-spin states, respectively. At zero bias, as shown in Fig. 5(e), no spin band overlap can be detected, and thereby no transmission peak occurs. However, when a negative bias is applied, the β-spin band in two electrodes approaches gradually and final overlapping, for example, at -0.2 V, as shown in Fig. 5(d), both of them have already a large overlap in the BW and generate a broaden and high transmission peak, thus a large β-spin current can emerge, while α-spin band in two electrodes goes far away gradually with bias, leading to a negligible small α-spin current. Under positive bias, behaviors for α (β)-spin state are similar to those for β(α)-spin state under negative bias, for example, at 0.2 V, as shown in Fig. 5(f), only α-spin band overlap occurs in the BW and creates a broaden and high transmission peak, and thus only a large α-spin current under positive bias is derived. Remarkably, these obtained results are entirely consistent with those in Fig. 4.

**Figure 6:**
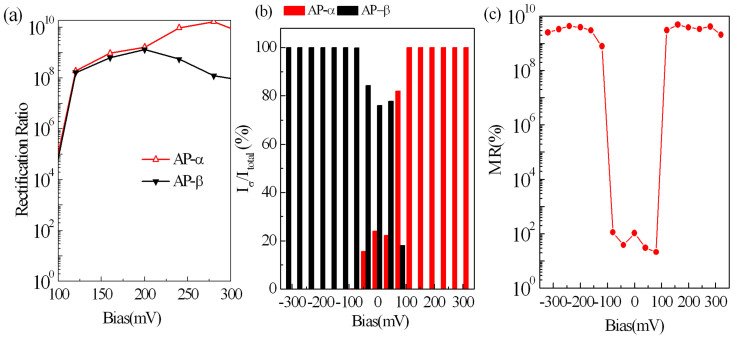
The spin-independent rectification ratio (a) and spin polarization (b) in AP configuration as well as magnetoresistace (c) for STGNR(5,3). The high-performance dual spin diode effect with a rectification ratio about 10^10^ and the dual spin-filtering effect with the perfect (100%) spin polarization, as well as a giant magnetoresistace (GMR) value approaching 10^10^%, can be achieved.

To quantify the STD characteristics of STGNRs, we define the spin-independent rectification ratio as 
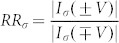
 (σ = α, β), spin polarization as 
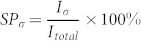
 (σ = α, β), and magnetoresistace as 
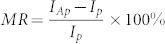
. The calculated results are shown in Fig. 6. As can be seen, an unexpectedly high rectification ratio, up to 10^10^, can be reached in AP configuration, as shown in Fig. 6(a), which is a forward rectification for α-spin state and an inverse rectification for β-spin state. This rectification ratio is a much larger value as compared to that for a ZGNR diode (~10^5^)^6^ and macroscopic p-n junction diodes (10^5^ ~ 10^7^), this means that the STGNR can act as an excellent dual spin diode. And also, the perfect (100%) spin polarization is achieved under a slightly higher bias (≥0.1 V) in AP configuration regardless of a bias being positive or negative, as shown in Fig. 6(b), serving as a dual spin-filtering device, namely, by the selection of bias polarity, we can obtain an electron flow with different spin directions. More importantly, one can see that, by a switching magnetic field applied on STGNRs, a highly effective spin-valve device is likely to be realized, which displays a giant magnetoresistace (GMR) approaching 10^10^%, which is three orders magnitude higher than that predicted based on the ZGNRs^5^ and six orders magnitude higher than previously reported experimental values for the MgO tunnel junction^28^,^29^,^30^.

## Discussion

Spintronics and magnetic device properties of STGNRs are investigated theoretically. It is found that at a FM ground state the STGNRs always feature the typical properties of bipolar magnetic semiconductors. And their Curie temperature *T_c_* is much higher as compared with that for ZGNRs. More importantly, the dual spin-filtering effect with the perfect (100%) spin polarization and high-performance dual spin diode effect with a rectification ratio about 10^10^ can be achieved, which is a much larger value as compared to that for a ZGNR diode (~10^5^) and macroscopic p-n junction diodes (10^5^ ~ 10^7^). Particularly, a highly effective spin-valve device is likely to be realized, which displays a giant magnetoresistace (GMR) approaching 10^10^% that is three orders magnitude higher than the value predicted based on the ZGNRs and six orders magnitude higher than previously reported experimental values for the MgO tunnel junction. These distinctive features can be attributed to their unique band overlap pattern for two electrodes and particular sensitivity to a switching magnetic field. Our findings suggest that STGNRs have a promising performance for developing STDs.

As we know, there exist two types of hybridizations, sp^2^- and sp^3^-hybridizations, for edge carbon atoms of GNRs. Under a lower hydrogen-concentration, the usual edge structure is generally regarded to be the sp^2^ hybridized mono-hydrogen termination. Therefore, mono-hydrogen terminated structures for GNRs are presented and investigated in most of literatures. In this present work, we also research on this type of termination for STGNRs. In fact, edge structure of GNRs is very diversified, such as dihydrogen termination^31^, edge reconstruction^32^, Z_211_ passivated edge^32^, edge defects^32^, and other chemical modifications, which might have an impact on magnetism. These complicated cases will be studied in our further works. Additionally, it might be difficult in achieving the required atomic precision for fabricating STGNRs in the current experiments, but theoretical modeling to understand the magnetic structure and magnetic transport properties of ideal STGNRs is very necessary.

Finally, we would like to point out that exchange -correlation functionals for DFT used in our work might underestimate the band gap of AGNRs compared with other algorithms, such as the GW method^33^. Therefore, a larger spin splitting energy gap *E_g_* might occur if using other more exact methods for calculations. In our work, the spin polarization and spin diode effect as well as the spin-valve device effect are all closely related to the spin splitting energy gap, which would lead to need a higher threshold voltage to start spintronic device effects.

## Methods

The geometric optimization as well as calculations of the electronic structure and transport are performed by using the spin-polarized DFT combined with the non-equilibrium Green's function(NEGF) method^34^,^35^. We employ Troullier-Martins norm-conserving pseudopotentials to represent the atom core and linear combinations of local atomic orbitals to expand the valence states of electrons. The spin-dependent generalized gradient approximation (SGGA) is used as the exchange–correlation functional is used as the exchange–correlation functional. The wave function is expanded by a single-zeta plus polarization (SZP) basis for H atoms, and double-zeta plus polarization (DZP) basis for other atoms. The k-point sampling is 1, 1, and 150 in the x, y, and z directions, respectively, where the z is the period direction of nanoribbon, and the cut off energy is set to 200 Ry. For all models studied, a 15Å vacuum slab is used to eliminate interaction between the models, and all calculation was performed after the geometries are optimized until all residual forces on each atom are smaller than 0.05 eV/Å. Once the convergence in self-consistency calculations is achieved, the spin-polarized current through a device is computed by the Landauer-like formula^36^. In our calculations, the average Fermi level, an average value of the chemical potential of the left and right electrodes, is set as zero.

## Author Contributions

Device design and theoretical analysis were performed by Z. Zg. Calculations for Electronic and magnetic structures, transmission spectra, and the I-V characteristics were performed mainly by D. W. and Secondarily by Z. Zu and B. L. All the authors discussed the results and wrote the manuscript.
